# Penicillin Allergy Labels and High-risk Antibiotic Prescribing Among Incarcerated Individuals Receiving Antibiotics Across Four US Carceral Systems

**DOI:** 10.1093/ofid/ofag128

**Published:** 2026-03-03

**Authors:** Samuel Wilk, Kap Sum Foong, Rachel Tam, Larissa Grigoryan, Lindsay Taylor, Lara B Strick, Rachel Sandler Silva, Alysse Wurcel

**Affiliations:** Tufts University School of Medicine, Boston, Massachusetts, USA; Division of Geographic Medicine and Infectious Diseases, Department of Medicine, Tufts Medical Center,Boston, Massachusetts, USA; Section of General Internal Medicine, Boston Medical Center, Boston, Massachusetts, USA; Department of Family and Community Medicine, Baylor College of Medicine, Houston, Texas, USA; Department of Medicine, University of Wisconsin School of Medicine and Public Health, Madison, Wisconsin, USA; Division of Allergy and Infectious Diseases, Department of Medicine, University of Washington, Seattle, Washington, USA; Washington State Department of Corrections, Tumwater, Washington, USA; Department of Medicine, University of Minnesota School of Medicine, Minneapolis, Minnesota, USA; Department of Medicine and Jail Health Services, Hennepin Healthcare System, Minneapolis, Minnesota, USA; Section of General Internal Medicine, Boston Medical Center, Boston, Massachusetts, USA

**Keywords:** antimicrobial stewardship, carceral setting, incarcerated populations, penicillin allergy, penicillin allergy delabeling

## Abstract

**Background:**

Optimization of antibiotic prescribing is critical to reducing antimicrobial resistance, yet antimicrobial stewardship programs remain relatively uncommon in carceral settings. Despite the disproportionate health burden faced by incarcerated populations, limited data exist on the prevalence of penicillin allergy labels (PALs) in these sites. This study examined the carceral systems in 4 states (Maine, New Hampshire, Washington, and Minnesota's Hennepin County Jail) and determined the prevalence of PALs, their demographic predictors, and the relationship between PALs and the prescription of high-risk antibiotics for *Clostridioides difficile* infection (CDI)—as defined by the National Healthcare Safety Network.

**Methods:**

We conducted a retrospective cohort study using the de-identified data from 4 carceral systems, restricted to incarcerated people who received at least 1 antibiotic prescription between 2020 and 2023. We performed univariate and multivariate logistic regression analyses, adjusting for age, sex, race, and ethnicity. We reported adjusted odds ratios (aORs) with 95% confidence intervals (CIs).

**Results:**

The final study cohort consisted of 10 603 individuals, with a PAL prevalence of 10.2%. Individuals with PALs were less likely to be male (aOR, 0.462; 95% CI, 0.355–0.601) or Hispanic (aOR, 0.500; 95% CI, 0.272–0.920) and more likely to be White non-Hispanic (aOR, 1.458; 95% CI, 1.075–1.978), or American Indian/Alaska Native (aOR, 2.466; 95% CI, 1.641–3.707). PALs were significantly associated with increased odds of receiving high-risk antibiotics for CDI (aOR, 2.412; 95% CI, 1.893–3.074).

**Conclusions:**

Our findings highlight the need for targeted antimicrobial stewardship and penicillin allergy de-labeling efforts among incarcerated individuals needing antibiotics.

## Teaser

In a multi-state study of incarcerated individuals receiving antibiotics, 10% had a penicillin allergy label, which was associated with more than twice the odds of high-risk C. difficile-associated antibiotic use. Correctional health systems should be included in antimicrobial stewardship and penicillin allergy delabeling to improve care and equity.

Antimicrobial stewardship curbs the use of overly broad and inappropriate antibiotics with the goal of decreasing bacterial resistance. One key strategy of antimicrobial stewardship is removal of incorrect penicillin allergy labels (PALs). Penicillin and other beta-lactam antibiotics are among the most preferred, cost-efficient, and safest treatments for common bacterial infections [1–3]. Approximately 10% of the United States population has a PAL; however, studies show that >90% of these labels are inaccurate [4, 5]. Mislabeling may arise from nonallergic adverse effects, vague or undocumented reactions, or a remote history of mild symptoms such as rash [4, 6]. In addition, a waning immunoglobulin response over time can make a historic allergy no longer relevant [4]. Individuals with PALs are more likely to receive alternative antibiotics that are broader in spectrum and associated with more significant adverse effects [4, 7–10]. These broad-spectrum and second-line antibiotics are also associated with suboptimal clinical outcomes for infection, *Clostridioides difficile* infection (CDI), longer hospital stays, and higher health care costs [5, 11]. Professional societies, such as the American Academy of Allergy, Asthma and Immunology and the Infectious Diseases Society of America, recommend proactive evaluation of PALs to identify individuals for penicillin allergy de-labeling [12].

Jails and prisons have long been at the epicenter of infectious epidemics due to their position at the intersection of disparities [13]. Approximately 1.9 million people in the United States are currently incarcerated, and 4.9 million have been formerly incarcerated [14]. The antimicrobial stewardship infrastructure is not common in carceral health care systems [15]. This is especially dangerous in jails and prisons, where the structural characteristics themselves promote infectious epidemics as seen during the coronavirus disease 2019 pandemic as well as with previous spread of methicillin-resistant *Staphylococcus aureus* [16, 17]. In addition, the incarcerated population is often more medically vulnerable due to higher rates of pre-incarceration poverty and comorbid conditions (eg, mental health issues, addiction, trauma), with reduced access to both preventative and acute care compared with the community. Thus, the largely unregulated antimicrobial prescribing practices that occur in these facilities risk the rapid spread of infection, especially multidrug-resistant infections [13]. Exacerbating this risk is the lack of proper resourcing of infection prevention programs to address multidrug-resistant organisms when they do emerge. Most of the nearly 2 million incarcerated people in the United States do not have a stewardship program to protect them from antimicrobial resistance, and carceral health systems lack clear accreditation standards that provide the incentive to prevent antibiotic misuse, which subsequently impacts community health systems.

There is a paucity of quality improvement and research literature examining interventions to decrease PALs in the carceral setting. The goal of this study was to evaluate the prevalence and demographic predictors of PALs and examine their association with the prescription of high-risk antibiotics for CDI in 1 county and 3 state carceral systems. Understanding these factors may inform future antibiotic stewardship and penicillin allergy de-labeling efforts in carceral settings. Because the clinical consequences of PALs arise primarily at the point of antibiotic prescribing, this study focuses on incarcerated individuals who received antibiotics. This approach allows for evaluation of antibiotic selection and exposure to high-risk antibiotics for CDI, but not estimation of population-level PAL prevalence.

## METHODS

We conducted a retrospective cohort study in partnership with clinicians at the Washington Department of Corrections (DOC), New Hampshire DOC, Maine DOC, and Hennepin County Jail (Minneapolis, MN, USA). Each site provided de-identified electronic health record data on all individuals who received at least 1 course of antibiotics during a fixed 1- to 2-year span between 2020 and 2023 while incarcerated. The variability in dates reflected the timing of approaching each of the centers for data and the feasibility of collecting the data. The data underlying this article cannot be shared publicly due to restrictions in data use agreements. The data will be shared on reasonable request to the corresponding author. Data collected included demographics, allergy documentation, and antibiotic prescriptions. Race and ethnicity were reported as a single combined variable. Penicillin allergy was defined as a documented allergy to penicillin, amoxicillin, amoxicillin-clavulanate, ampicillin, ampicillin-sulbactam, or piperacillin-tazobactam. High-risk antibiotics for CDI were defined according to National Healthcare Safety Network (NHSN) criteria, which include third- and fourth-generation cephalosporins, fluoroquinolones, and clindamycin (Supplementary Material 1) [18].

### Statistical Analysis

Demographic, allergy, and antibiotic prescription data were summarized using frequencies (with percentages) for categorical variables and means (with SDs) for continuous variables. We performed univariate and multivariate logistic regression analyses to evaluate 2 outcomes: (1) demographic predictors of a documented PAL and (2) the association between PAL and receipt of high-risk antibiotics for CDI. We also conducted exploratory secondary analyses, specifically to identify predictors of fluoroquinolone and clindamycin prescribing individually, because those are typically the most commonly overprescribed high-risk antibiotics. These exploratory models were adjusted for age, sex, race/ethnicity, and receipt of other non-high-risk antibiotics. In addition to demographic variables, we chose to adjust for non-high-risk antibiotics in order to see if people who were being prescribed other lower-risk antibiotics were also being prescribed high-risk antibiotics as well. All analyses were conditional on antibiotic exposure and were not intended to estimate the prevalence of PALs in the overall incarcerated population.

All variables from univariate comparisons were incorporated within a final, adjusted multivariable model. We report adjusted odds ratios (aORs) with 95% CIs. Statistical analyses were performed using SPSS, version 25 (IBM, Armonk, NY, USA). Two-sided *P* values were considered statistically significant at ≤.05 in the multivariate analyses. This study was reviewed by the Institutional Review Board (IRB) of Tufts Medical Center, which deemed the study to be low risk and therefore exempt from full review, including from the individual states' DOCs.

## RESULTS

Of the 10 603 individuals who received antibiotics while incarcerrated, 1079 (10.2%) had a documented PAL. In the PAL group, 31.9% were female, 61.9% White, 18.4% Black, and 4.2% Hispanic. In the non-PAL group, 16.7% were female, 54.9% White, 24.6% Black, and 11.0% Hispanic (Table 1). Race and ethnicity data were unavailable for individuals from Maine.

**Table 1.** Demographic Characteristics and Receipt of High-risk Antibiotics for *Clostridioides difficile* Infection Among Incarcerated Individuals who Received at Least One Course of Antibiotics Across Four States

Demographic Characteristic	No. of Individuals (%)		
	Total Cohort, n = 10 603	With PAL, n = 1079	Without PAL, n = 9524
Age, mean (SD), y	43.5 (12.1)	43.8 (12.4)	43.5 (12.1)
Sex			
Female	1935 (18.2)	344 (31.9)	1591 (16.7)
Male	8668 (81.8)	735 (68.1)	7933 (83.3)
Race/ethnicity^a^			
Black (African American or African)	1632 (24.3)	57 (18.4)	1575 (24.6)
White (Caucasian, non-Hispanic)	3702 (55.2)	192 (61.9)	3510 (54.9)
Hispanic (Latino)	718 (10.7)	13 (4.2)	705 (11.0)
American Indian (Native American) or Alaskan Native	493 (7.4)	46 (14.8)	447 (4.7)
Asian	117 (1.7)	2 (0.6)	115 (1.8)
Unknown	43 (0.6)	0	43 (0.7)
Receipt of high-risk antibiotics for CDI^b^	2148 (20.3)	377 (34.9)	1771 (18.6)

Abbreviations: CDI, *Clostridiodes difficile* infection; PAL, penicillin allergy label.

^a^Only included 6705; 3898 individuals from Maine did not have data on race/ethnicity.

^b^High-risk antibiotics for CDI include third- and fourth-generation cephalosporins, fluoroquinolones, and clindamycin.

In the final multivariate logistic regression model, demographic factors associated with a documented PAL include White non-Hispanic (aOR, 1.458; 95% CI, 1.075–1.978) and American Indian/Alaska Native (aOR, 2.466; 95% CI, 1.641–3.707). Male sex (aOR, 0.462; 95% CI, 0.355–0.601) and Hispanic ethnicity (aOR, 0.500; 95% CI, 0.272–0.920) were associated with lower odds of having a PAL (Table 2).

**Table 2.** Predictors of Penicillin Allergy Label in a Cohort of Incarcerated People who Received Antibiotics Across Four States

Predictor	Unadjusted OR (95% CI)	*P* Value	Adjusted OR^a^ (95% CI)	*P* Value
Age	1.002 (0.997–1.007)	.402	1.003 (0.993–1.031)	.548
Sex				
Female	Reference		Reference	…
Male	0.429 (0.373–0.492)	**<.001**	0.462 (0.355–0.601)	**<.001**
Race/ethnicity				
Black (African American or African)	Reference		Reference	…
White (Caucasian, non-Hispanic)	1.511 (1.118–2.043)	**.007**	1.458 (1.075–1.978)	**.015**
Hispanic (Latino)	0.510 (0.277–0.937)	**.030**	0.500 (0.272–0.920)	**.026**
American Indian (Native American) or Alaskan Native	2.844 (1.901–4.252)	**<.001**	2.466 (1.641–3.707)	**<.001**
Asian	0.481 (0.116–1.993)	.313	0.485 (0.117–2.013)	.319

Significant *P* values are bolded.

Abbreviation: OR, odds ratio.

^a^Adjusted for age, sex, race/ethnicity.

A total of 13 823 antibiotic prescriptions were recorded, averaging 1.3 prescriptions per person. The most commonly prescribed antibiotics were penicillins (28.9%), tetracyclines (21.1%), and trimethoprim-sulfamethoxazole (12.2%). Despite their allergy label, 12.6% of prescriptions received by patients with a PAL were β-lactams. In comparison, however, β-lactams accounted for 55.0% of prescriptions received by individuals without a PAL (Supplementary Table 1). High-risk antibiotics for CDI were prescribed in 34.9% of individuals with a PAL compared with 18.6% without a PAL (Table 1). In multivariable analysis adjusting for age, sex, and race/ethnicity, PAL was associated with significantly higher odds of receipt of antibiotics categorized as high risk for CDI (aOR, 2.412; 95% CI, 1.893–3.074) (Table 3).

**Table 3.** Predictors of Receipt of High-risk Antibiotics for *Clostridioides difficile* Infection in a Cohort of Incarcerated People who Received Antibiotics Across Four States

Predictor	Unadjusted OR (95% CI)	*P* Value	Adjusted OR (95% CI)^a^	*P* Value
Age	1.012 (1.008–1.015)	**<.001**	1.016 (1.011–1.020)	**<.001**
Sex				
Female	Reference		Reference	…
Male	0.888 (0.788–1.001)	.053	1.262 (1.057–1.506)	**.010**
Race/ethnicity				
Black (African American or African)	Reference		Reference	…
White (Caucasian, non-Hispanic)	1.305 (1.128–1.510)	**<.001**	1.213 (1.045–1.407)	**.011**
Hispanic (Latino)	0.878 (0.697–1.107)	.272	0.918 (0.727–1.159)	.472
American Indian (Native American) or Alaskan Native	1.007 (0.779–1.304)	.955	0.993 (0.764–1.289)	.956
Asian	0.948 (0.582–1.545)	.830	0.956 (0.585–1.560)	.856
Other	1.490 (0.743–2.988)	.262	1.359 (0.674–2.074)	.391
PAL	2.351 (2.053–2.692)	**<.001**	2.412 (1.893–3.074)	**<.001**

Significant *P* values are bolded.

Abbreviations: OR, odds ratio; PAL, penicillin allergy label.

^a^Adjusted for age, sex, race/ethnicity, and PAL.

**Figure 1.**
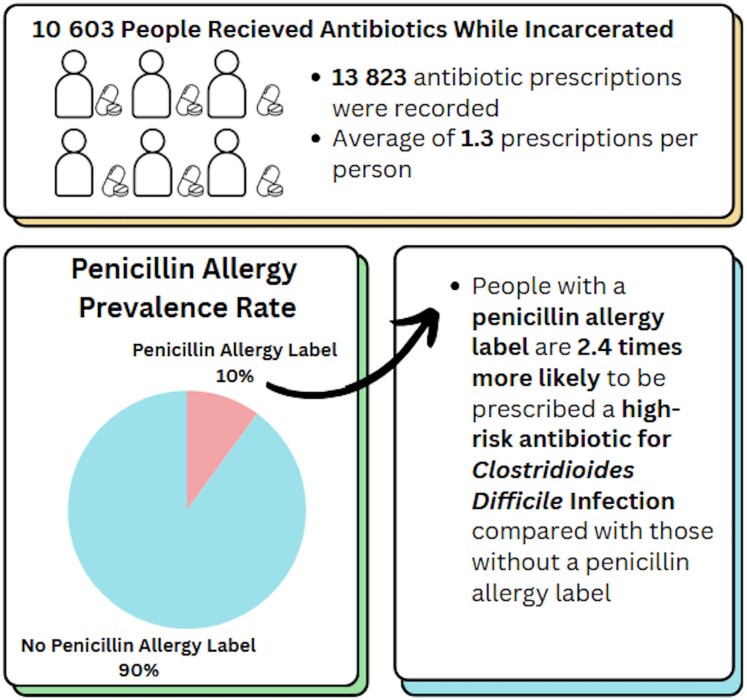
Penicillin allergy label and *Clostridioides difficile* infection infographic. Figure 1 presents information about antibiotic prescribing and penicillin allergy within carceral settings.

In exploratory secondary analyses, PAL was also associated with increased odds of receiving fluoroquinolones (aOR, 2.251; 95% CI, 1.407–3.600) (Supplementary Table 2) and clindamycin (aOR, 1.632; 95% CI, 1.103–2.414) (Supplementary Table 3), after adjustment for demographic covariates and receipt of non-high-risk antibiotics. We have summarized these findings in the form of an infographic (Figure 1) for the intended audience of public health officials, administrative staff in correctional facilities, and health care providers.

## DISCUSSION

In this multistate study of individuals who received antibiotics while incarcerrated, 10.2% had a documented PAL, a proportion lower than that reported in other health care settings [19, 20]. By design, our analyses were restricted to individuals who received antibiotics, as the clinical impact of PALs occurs primarily at the time of antibiotic prescribing. Consistent with prior studies evaluating the downstream consequences of antibiotic allergy labels, this approach conditions analyses on antibiotic exposure rather than estimating population-level allergy prevalence [19, 20]. Within this treated cohort, the presence of a PAL was associated with increased exposure to antibiotic classes categorized as high risk for CDI. As we lacked data on the infectious indications and microbiologic susceptibilities, we cannot determine whether individual antibiotic selections were appropriate or chosen specifically because of a documented PAL. Even when antibiotic selection is clinically appropriate and informed by resistance concerns, understanding how PALs shape overall antibiotic exposure remains relevant in carceral settings, where opportunities for allergy verification, de-labeling, and antibiotic optimization are limited.

The downstream adverse consequences of repeated exposure to high-risk agents may also be amplified in carceral settings as structural vulnerabilities, resource limitations, and high baseline risk for infectious complications amplify the potential downstream harms of antibiotic-related adverse events, including CDI [14, 21]. Consistent with previous literature in the community, White race was associated with higher odds of PALs among incarcerated individuals, likely reflecting differential access to health care and increased opportunities for allergy labeling through repeated health care encounters before incarceration [21–25]. Higher prevalence of PALs in White populations can be noted as early as childhood, where studies have shown that racial differences in antibiotic prescriptions lead to non-Hispanic White children having greater exposure to penicillin and thus a greater likelihood to develop rashes and receive a PAL. In addition to these differences in health care system interactions by race and ethnicity, the fact that the great majority of dermatological resource books display images on rashes on lighter skin tone may facilitate easier recognition in White children [21, 26].

Notably, we identified significantly higher odds of PALs among American Indian and Alaskan Native individuals. This unexpected finding warrants further investigation. Native Americans are the largest racial or ethnic group per capita in the United States prison system [27]. We have some preliminary hypotheses about the connection between PAL and Native American identity. The restricted formularies for Indian Health Service clinics, the National Core Formulary includes only 23 antibiotic listings including topical and ophthalmic formulations [28], may result in more frequent use of β-lactams, leading to more PALs. This might be caused by infections that are nonresponsive to β-lactams, which leads to the misinterpretation of treatment failure and disease progression as an allergic reaction. We also wonder how increased rates of living in rural areas [29] with financial and structural barriers to health care may influence an increased rate of PALs [30, 31]. There is some work providing education around antibiotics to Native populations [32], but more work needs to be done, especially in people who are Native and incarcerated.

Female sex was also found to be associated with PAL. Higher odds of PALs in women are well documented in the community and are again likely due to increased antibiotic prescriptions and usage. Women are 27% more likely to receive an antibiotic prescription than men and receive 25% more antibiotic courses than men, from a combination of increased medical visits and more inappropriate prescribing [33]. Uncomplicated urinary tract infections (UTIs), seen more commonly in women, are one of the most common bacterial infections, and thus UTI treatment (both appropriate and inappropriate) plays an important role in increased antibiotic usage [34]. There are several bacterial infections more common in carceral settings that necessitate antibiotic treatment, such as the sexually transmitted infections (STIs) *Chlamydia trachomatis, Neiseeria gonorrhea,* and syphilis, especially among women [35, 36]. In addition, women are more likely to self-report drug allergies, leading to greater prevalence of PAL in this group [37]. While women represent just 10% of the incarcerated population, the rate of incarceration for women has grown at twice the rate of men [38].

Penicillins and β-lactams are among the safest classes of antibiotics; therefore, limiting their use increases the risk of receiving other, higher-risk classes. However, as we did not have data on the infectious indication being treated in this cohort, we were not able to assess the appropriateness of individual antibiotic prescriptions. Certain infections common in carceral settings, such as community-associated methicillin-resistant *Staphylococcus aureus*, may appropriately warrant treatment with clindamycin or trimethoprim-sulfamethoxazole regardless of penicillin allergy status. Nevertheless, the higher prevalence of infections for which β-lactams remain first-line therapy, such as syphilis and gonorrhea, poses challenges for patients with documented PALs, as alternative treatments are often longer, more complex, more prone to failure, and increasingly compromised by antimicrobial resistance [39, 40]. At the community level, the emphasis remains on penicillin allergy de-labeling initiatives for people with infections such as a UTIs to allow for best course treatment and restrict further resistance [41, 42].

Development of new and targeted methods for penicillin allergy de-labeling in low-resource and difficult-to-reach settings is important to ensure that antibiotic stewardship reaches all facets of the health care system, including carceral settings. Historically, de-labeling has been conducted by allergists in specialty clinics and hospital wards. In the community setting, the practice has been spreading to include primary care clinics thanks to the development of safer and more accessible de-labeling protocols that include comprehensive allergy history assessments, risk stratification tools, and direct oral drug challenges [15, 43]. Despite greater access in the community, there are several barriers preventing implementation of penicillin allergy de-labeling processes in jails and prisons. Some of these barriers are the same as those that occur in long-term care facilities: resource constraints, staffing shortages, high burnout, regulatory requirements, and limited research [15]. Yet other barriers are unique to the carceral health system itself.

For example, a rapid response to an acute allergic reaction during a de-labeling protocol looks different than it would in a community health setting. Many carceral facilities are in remote locations, access to emergent medications like epinephrine may be limited due to regulations around sharps, and the numerous steps required to move an individual to a higher level of care may have downstream ramifications for de-labeling protocols. Despite these barriers, penicillin allergy de-labeling in carceral health systems is crucial to preventing the spread of antimicrobial resistance and is critical in preventing CDI. The complex and under-resourced carceral system cannot afford the risks associated with such infections and thus must develop resource-conscious interventions that reduce inaccurate PALs and minimize the prescription of high-risk antibiotics for CDI. While such interventions may be more easily established in prisons than in jails due to longer durations of stay, they are equally important across the 2 settings and will play an important role in equalizing care between the carceral and community settings.

Also, the lack of integration of carceral and community health care records creates a barrier to communication, such that even when penicillin allergy de-labeling occurs in the community, the updated information may not carry over to the carceral health record. With lower health literacy rates in prisons and jails, patients may be less likely to report the change in their record themselves. To date, the efforts to raise awareness among patients and promote screening among providers for inaccurate PALs have focused on community settings. Targeted education for correctional staff and individuals who are incarcerated is greatly needed to lead the necessary practice changes to increase penicillin allergy de-labeling.

This study has several limitations. Our study was restricted to individuals who received antibiotics while incarcerated, and the findings may not be generalizable to the entire incarcerated population. Individuals prescribed antibiotics may differ systematically from those who not prescribed antibiotics, including differences in health care access, duration of incarceration, clinical complexity, and health-seeking behaviors. These factors may confound observed associations between PALs and antibiotic selection. However, because the primary outcome, receipt of high-risk antibiotics for CDI, occurred mostly among individuals exposed to antibiotics, this study base was necessary to address the research question. Our study also lacked detailed clinical data on indications for antibiotic use, comorbidities, prescribing provider, and specific reaction types. As a result, we were unable to assess the appropriateness of individual antibiotic prescriptions and whether the antibiotic selection reflected penicillin allergy avoidance, resistance considerations, or both. Observed associations should therefore be interpreted as differences in antibiotic exposure patterns rather than evidence of inappropriate prescribing. These factors may confound the relationship between PAL and receipt of high-risk antibiotics for CDI. We also lack data on follow-up outcomes such as resolution of infection and need for further antibiotic prescriptions. Additionally, PALs were based on electronic health record documentation and not verified through clinical history or allergy testing, limiting our ability to distinguish between true and false labels. However, we assume that like the community, many PALs were not true. The data set does not capture multiple antibiotic allergies, such as cephalosporin allergies, that otherwise would influence antibiotic choice. We were unable to separate race and ethnicity due to how these data were reported, which may obscure important subgroup differences. Finally, the findings may not generalize beyond the participating carceral systems.

In conclusion, our findings emphasize the need to include carceral health systems in broader antimicrobial stewardship and penicillin allergy de-labeling strategies. Tailored, resource-conscious interventions, such as structured allergy history taking, clinical decision support, or point-of-care stratification, may offer feasible alternatives in low-resource settings. Addressing PALs among individuals who received antibiotics while incarcerated is not only a stewardship priority but also a matter of health equity.

## Supplementary Material

ofag128_Supplementary_Data

## Supplementary Data

Supplementary materials are available at *Open Forum Infectious Diseases* online. Consisting of data provided by the authors to benefit the reader, the posted materials are not copyedited and are the sole responsibility of the authors, so questions or comments should be addressed to the corresponding author.

## Notes

***Acknowledgments.*** We would like to acknowledge the support and research resources from Maine Department of Corrections, Hennepin Healthcare, New Hampshire Department of Corrections, and Washington Department of Corrections.

***Author contributions.*** Sam Wilk (writing, editing), Kap Sum Foong (writing, editing, analysis), Rachel Tam (writing, editing), Larissa Grigoryan (editing), Lindsay Taylor (editing), Lara Strick (editing), Rachel Silva (editing), Alysse Wurcel (concept generation, editing).
