# Supplementary material for: Penicillin Allergy Labels and High-risk Antibiotic Prescribing Among Incarcerated Individuals Receiving Antibiotics Across Four US Carceral Systems
Source: Open Forum Infect Dis. 2026 Mar 3;13(3):ofag128. doi: 10.1093/ofid/ofag128 (PMC13014467; doi:10.1093/ofid/ofag128)
Supplement: ofag128_Supplementary_Data [file ofag128_supplementary_data.zip › Supplementary_material_1.docx]

**Supplemental Material 1:** Adult Antibacterial agents posing the highest risk for *Clostridioides Difficile* Infection

Cefdinir

Cefepime

Cefixime

Cefotaxime

Cefpodoxime

Ceftazidime

Ceftriaxone

Ciprofloxacin

Clindamycin

Gemifloxacin

Levofloxacin

Moxifloxacin
