# Supplementary material for: Penicillin Allergy Labels and High-risk Antibiotic Prescribing Among Incarcerated Individuals Receiving Antibiotics Across Four US Carceral Systems
Source: Open Forum Infect Dis. 2026 Mar 3;13(3):ofag128. doi: 10.1093/ofid/ofag128 (PMC13014467; doi:10.1093/ofid/ofag128)
Supplement: ofag128_Supplementary_Data [file ofag128_supplementary_data.zip › Supplementary_Table_1.docx]

**Supplementary Table 1:** Antibiotic classes prescribed to people in the cohort.

| **Antibiotic class** | **Number of Prescriptions (%)** | | |
| --- | --- | --- | --- |
|  | **Total prescriptions** | **Prescriptions for People With PAL n=1079** | **Prescriptions for People Without PAL n=8663** |
| Penicillin | 3995 | 34 (3.2) | 3961 (41.6) |
| First-generation cephalosporin | 912 | 51 (4.7) | 861 (9.0) |
| Second-generation cephalosporin | 57 | 15 (1.4) | 42 (0.4) |
| Third-generation cephalosporin | 442 | 36 (3.3) | 406 (4.3) |
| Fourth generation cephalosporin | 4 | 0 | 4 (0.1) |
| Carbapenem | 5 | 0 | 5 (0.1) |
| TMP-SMX | 1698 | 196 (18.2) | 1502 (15.8) |
| Macrolide | 1014 | 100 (9.3) | 914 (9.6) |
| Tetracycline | 2915 | 345 (32.0) | 2570 (27.0) |
| Fluoroquinolone | 556 | 93 (8.6) | 463 (4.9) |
| Clindamycin | 1267 | 266 (24.7) | 1001 (10.5) |
| Metronidazole | 789 | 76 (7.0) | 713 (7.5) |
| Linezolid | 8 | 0 | 8 (0.1) |
| Nitrofurantoin | 90 | 20 (1.9) | 70 (0.7) |
| Aminoglycoside | 9 | 3 (0.3) | 6 (0.1) |
| Vancomycin | 57 | 3 (0.3) | 54 (0.6) |
| Fosfomycin | 4 | 1 (0.1) | 3 (0.1) |
| Daptomycin | 1 | 0 | 1 (0.1) |

PAL, penicillin allergy label
