# Supplementary material for: Penicillin Allergy Labels and High-risk Antibiotic Prescribing Among Incarcerated Individuals Receiving Antibiotics Across Four US Carceral Systems
Source: Open Forum Infect Dis. 2026 Mar 3;13(3):ofag128. doi: 10.1093/ofid/ofag128 (PMC13014467; doi:10.1093/ofid/ofag128)
Supplement: ofag128_Supplementary_Data [file ofag128_supplementary_data.zip › Supplementary_Table_2.docx]

**Supplementary Table 2:** Predictors of receipt of fluoroquinolones in a cohort of incarcerated people who received antibiotics across four states

| **Predictor** | **Unadjusted OR (95% CI)** | ***p* value** | **Adjusted OR* (95% CI)** | ***p* value** |
| --- | --- | --- | --- | --- |
| Age | 1.051 (1.045-1.057) | **<0.001** | 1.058 (1.048-1.067) | **<0.001** |
| Sex |  |  |  |  |
| Female | Reference |  | Reference |  |
| Male | 0.758 (0.617-0.931) | **0.008** | 2.400 (1.451-3.967) | **<0.001** |
| Race/Ethnicity | | | | |
| Black (African American or African) | Reference |  | Reference |  |
| White (Caucasian, Non-Hispanic) | 1.464 (1.102-1.946) | **0.009** | 1.315 (0.933-1.853) | 0.117 |
| Hispanic (Latino) | 0.942 (0.596 – 1.489) | 0.798 | 1.102 (0.663 – 1.916) | 0.732 |
| American Indian (Native American) or Alaskan Native | 0.914 (0.537 – 1.555) | 0.739 | 0.995 (0.528 – 1.877) | 0.988 |
| Asian | 1.303 (0.552 – 3.074) | 0.545 | 1.257 (0.419 – 3.371) | 0.684 |
| PAL | 1.846 (1.463 – 2.328) | **<0.001** | 2.251 (1.407 – 3.600) | **<0.001** |
| Prescription of non-fluoroquinolone antibiotics | 0.002 (0.001 – 0.002) | **<0.001** | 0.005 (0.003 – 0.009) | **<0.001** |

PAL, penicillin allergy label; OR, odds ratio; CI, confidence interval

*adjusted for age, sex, race/ethnicity, PAL, and prescription of non-fluoroquinolone antibiotics. Significant p-values are bolded.
