# Supplementary material for: Penicillin Allergy Labels and High-risk Antibiotic Prescribing Among Incarcerated Individuals Receiving Antibiotics Across Four US Carceral Systems
Source: Open Forum Infect Dis. 2026 Mar 3;13(3):ofag128. doi: 10.1093/ofid/ofag128 (PMC13014467; doi:10.1093/ofid/ofag128)
Supplement: ofag128_Supplementary_Data [file ofag128_supplementary_data.zip › Supplementary_Table_3.docx]

**Supplementary Table 3:** Predictors of receipt of clindamycin in a cohort of incarcerated people who received antibiotics across four states

| **Predictor** | **Unadjusted OR (95% CI)** | ***p* value** | **Adjusted OR* (95% CI)** | ***p* value** |
| --- | --- | --- | --- | --- |
| Age | 0.995 (0.990-1.000) | 0.032 | 1.006 (0.999-1.014) | 0.098 |
| Sex |  |  |  |  |
| Female | Reference |  | Reference |  |
| Male | 0.911 (0.786-1.058) | 0.221 | 1.403 (1.056-1.865) | 0.020 |
| Race/Ethnicity | | | | |
| Black (African American or African) | Reference |  | Reference |  |
| White (Caucasian, Non-Hispanic) | 2.099 (1.712-2.573) | **<0.001** | 2.377 (1.821-3.103) | **<0.001** |
| Hispanic (Latino) | 1.282 (0.942 – 1.744) | 0.114 | 1.755 (1.204 – 2.559) | **0.003** |
| American Indian (Native American) or Alaskan Native | 1.094 (0.757 – 1.580) | 0.634 | 1.083 (0.666 – 1.762) | 0.748 |
| Asian | 1.127 (0.575 – 2.209) | 0.728 | 1.579 (0.723 – 3.453) | 0.252 |
| PAL | 2.786 (2.390 – 3.247) | **<0.001** | 1.632 (1.103 – 2.414) | **0.014** |
| Prescription of non-clindamycin antibiotics | 0.001 (0.001 – 0.002) | **<0.001** | 0.005 (0.003 – 0.008) | **<0.001** |

PAL, penicillin allergy label; OR, odds ratio; CI, confidence interval

*adjusted for age, sex, race/ethnicity, PAL, and prescription of non-clindamycin antibiotics Significant P values are bolded
